# Deep phenotyping reveals movement phenotypes in mouse neurodevelopmental models

**DOI:** 10.1186/s13229-022-00492-8

**Published:** 2022-03-12

**Authors:** Ugne Klibaite, Mikhail Kislin, Jessica L. Verpeut, Silke Bergeler, Xiaoting Sun, Joshua W. Shaevitz, Samuel S.-H. Wang

**Affiliations:** 1 Department of Organismic and Evolutionary Biology, Harvard University, 52 Oxford St, 02138 Cambridge, MA USA; 2 Princeton Neuroscience Institute, Princeton University, Washington Rd, 08544 Princeton, NJ USA; 3 Department of Physics, Lewis-Sigler Institute for Integrative Genomics, Princeton University, Washington Rd, 08544 Princeton, NJ USA

**Keywords:** Autism, Cerebellum, Mouse, Pose estimation, Clustering, Behavior

## Abstract

**Background:**

Repetitive action, resistance to environmental change and fine motor disruptions are hallmarks of autism spectrum disorder (ASD) and other neurodevelopmental disorders, and vary considerably from individual to individual. In animal models, conventional behavioral phenotyping captures such fine-scale variations incompletely. Here we observed male and female C57BL/6J mice to methodically catalog adaptive movement over multiple days and examined two rodent models of developmental disorders against this dynamic baseline. We then investigated the behavioral consequences of a cerebellum-specific deletion in Tsc1 protein and a whole-brain knockout in Cntnap2 protein in mice. Both of these mutations are found in clinical conditions and have been associated with ASD.

**Methods:**

We used advances in computer vision and deep learning, namely a generalized form of high-dimensional statistical analysis, to develop a framework for characterizing mouse movement on multiple timescales using a single popular behavioral assay, the open-field test. The pipeline takes virtual markers from pose estimation to find behavior clusters and generate wavelet signatures of behavior classes. We measured spatial and temporal habituation to a new environment across minutes and days, different types of self-grooming, locomotion and gait.

**Results:**

Both Cntnap2 knockouts and L7-Tsc1 mutants showed forelimb lag during gait. L7-Tsc1 mutants and Cntnap2 knockouts showed complex defects in multi-day adaptation, lacking the tendency of wild-type mice to spend progressively more time in corners of the arena. In L7-Tsc1 mutant mice, failure to adapt took the form of maintained ambling, turning and locomotion, and an overall decrease in grooming. However, adaptation in these traits was similar between wild-type mice and Cntnap2 knockouts. L7-Tsc1 mutant and Cntnap2 knockout mouse models showed different patterns of behavioral state occupancy.

**Limitations:**

Genetic risk factors for autism are numerous, and we tested only two. Our pipeline was only done under conditions of free behavior. Testing under task or social conditions would reveal more information about behavioral dynamics and variability.

**Conclusions:**

Our automated pipeline for deep phenotyping successfully captures model-specific deviations in adaptation and movement as well as differences in the detailed structure of behavioral dynamics. The reported deficits indicate that deep phenotyping constitutes a robust set of ASD symptoms that may be considered for implementation in clinical settings as quantitative diagnosis criteria.

**Supplementary Information:**

The online version contains supplementary material available at 10.1186/s13229-022-00492-8.

## Background

The highly variable phenotypes associated with neurodevelopmental disorders such as autism spectrum disorder (ASD) present a challenge to classification. Diagnosis of ASD draws heavily on the identification of distinctive actions. Arising in early life, this disorder is defined by abnormal social interactions and communication, stereotyped repetitive behaviors and restricted interests [1, 2]. The intensity and manifestation of these traits can vary greatly between individuals. In addition to cognitive and social variation, persons on the autism spectrum also express variable degrees of deficit in movement and sensory response [3–5]. The entire range of variation is currently classified for treatment purposes as a single broad entity, ASD [6, 7].

For a highly heritable disorder such as autism, mice provide an attractive model because they open the possibility of studying the consequences of a particular genetic or environmental factor repeatedly among many individuals. Mouse models of autism and other disorders have been designed to reflect known risk factors and causes in humans (construct validity) [8, 9]. Models are often selected for investigation based on their putative behavioral similarities to the human disorder (face validity) and have traits that include perseveration, disrupted social preference and deficits in flexible learning. Face-valid traits can be observed in conjunction with other traits that are usually considered to be of secondary interest, such as sensory and motor deficits. Those phenotypes originate from the same genetic perturbation that produced the primary traits of interest, making them potentially useful for linking symptoms to underlying mechanisms.

Traditionally, mouse behavioral testing consists of discrete measurements such as location in a multi-arm maze or a three-chamber test apparatus [10, 11]. Such characterization omits both finer details of movement and higher-order complex behavioral motifs [12]. These details can now be extracted efficiently using modern computational methods in machine vision. Recent advances in automated tracking allow deep phenotyping of movement consisting of simultaneous tracking of body-centric joint and body part positions, $x-y$ position in an arena and task performance, thereby providing a multi-level view of behavior [13–16]. This allows the measurement of movement and cognitive/social features from a single dataset [17, 18]. We developed a method using such a system for semi-supervised behavioral classification in individual mice during spontaneous activity. Our method describes long-term structure and fine-scale kinematic details in repeated recordings. Body part identification is consistent within individual mice over multiple sessions in the open-field arena. From these detailed features, we used posture dynamical clustering [19–22] to quantify the entire repertoire of movement.

We established a description of a commonly used inbred strain of mice, C57BL/6J (‘Black6’), to characterize baseline behavior and to identify sex differences. Sex-dependent differences in gene regulation have been suggested to underlie the higher incidence of autism in males [23]. We then identified features of movement that were correlated with genetic background and specific genetic manipulations [10, 24, 25]. As a test of the method’s discriminatory power, we explored gene-knockout (KO) models of neurodevelopmental disorders that perturb either the whole brain (Cntnap2 KO) or are cerebellum-specific (L7-Tsc1 mutant). These two lines are well-studied monogenic strains used to investigate autism endophenotypes [26]. Cntnap2 KO mice have been reported as hyperactive and have been shown to display a mild change in gait, along with reduced time spent with a novel partner in social behavior assays [27, 28]. Mice with Purkinje cell-specific (L7) null mutation of *tuberous* *sclerosis* 1 (*Tsc*1) reportedly exhibit a variety of social deficits compared to wild-type littermates, along with changes in gait, increased time spent grooming and decreased behavioral flexibility [29–31]. Postural defects have been recently observed in many mouse models of autism [25], revealing an opportunity for deep phenotyping using machine vision methods. Both of these strains exhibit similar altered spontaneous and task behavior compared to WT when using coarse-grained metrics; we applied our deep behavioral phenotyping to test for distinct signatures of behavior under spontaneous non-task conditions.

## Methods

Experimental animals C57BL/6J male ($n = 60$) and female ($n=20$) were ordered from Jackson Laboratory (The Jackson Laboratory, Bar Harbor, ME) and acclimated from 6 to 20 days in the Princeton Neuroscience Institute vivarium before experimental procedures. Two mouse models were used to analyze autism-like endophenotypes.

$L7-Tsc1$: To test cerebellar modulation of naturalistic behaviors, a Purkinje cell degeneration model with a tuberous sclerosis 1 gene mutation was used [29, 32]; $L7^{Cre};Tsc1^{flox/flox}$. Initially, $Tsc1^{flox/flox}$ ($Tsc1^{tm1Djk}$/J, Jackson Laboratory stock 005680) mutant mice were crossed into *L*7/*Pcp*2 mice (B6.129-Tg(Pcp2-cre)2Mpin/J, Jackson Laboratory stock 004146) to create a Purkinje cell-specific mutation $L7^{Cre};Tsc1^{flox/+}$. The progeny used from this cross are control ($L7^{Cre}$;$Tsc1^{+/+}$), heterozygous ($L7^{Cre}$;$Tsc1^{flox/+}$) mice and mutant ($L7^{Cre}$;$Tsc1^{flox/flox}$) mice. Only male animals were used for behavior experiments. Mice are of mixed genetic backgrounds (C57Bl/6J, 129 SvJae and BALB/cJ).

*Cntnap*2: A knockout of *Cntnap*2 associated with cortical dysplasia–focal epilepsy, $Cntnap2^{-/-}$ (B6.129(Cg)- $Cntnap2^{tm1Pele}$/J, stock 017482) was bred to C57BL/6J (Jackson Laboratory stock 000664) male mice to obtain heterozygote ($Cntnap2^{+/-}$) progeny. These litters were then bred as a heterozygote strategy to obtain litters with wild type (WT, $Cntnap2^{+/+}$), full knockout (KO, $Cntnap2^{-/-}$) and heterozygote ($Cntnap2^{+/-}$) mice.

All animals were tested in adulthood (> 10 weeks of age) and housed with four littermates per cage in Optimice cages (Animal Care Systems, Centennial, CO). PicoLab Rodent Diet food pellets (LabDiet, St. Louis, MO) and drinking water were provided ad libitum. All mice were kept in a reverse light cycle (12 h:12 h, light:dark with switch at 7:30 am:pm) for behavioral testing.

### Open-field test recordings

Animals were placed in an open-field area measuring 45.7 x 45.7 cm and 30.5 cm in height with a transparent polycarbonate floor 60 cm above the floor and 60 cm below the ceiling. A Point Grey grayscale camera (12-bit grayscale, $1280 \times 1024$ pixel resolution at 80 Hz) was used to image animals from below. To avoid fixed and dynamic environmental factors, which may act as a confounding variables on mouse behavior [10], tests were carried out by five testers carefully following a protocol which involved weighing the mouse, acclimating mouse in a testing room with dim red light and placing the mouse into the arena. The open-field arena was illuminated with far-red 850 nm LEDs to prevent mice from seeing the transparent floor and to minimize anxiety triggered by bright illumination. The arena was placed 30 cm away from the walls and 15 cm away from the doors of ventilated soundproof box to prevent noise disturbance. Doors were kept closed during acquisition. Room temperature was fixed at $21\pm 2^\circ {\mathrm{C}}$. Each mouse was recorded for 20 min before being returned to group housing. Movies were saved as h5 files for further processing in MATLAB.

### Centroid tracking and clipping

Full-frame movies were imaged at $1280 \times 1024$ pixel resolution and contained the entire arena in the field of view. Mouse centroids were tracked and movies were clipped prior to performing joint tracking. Each movie was first sampled at random to generate a median image from 50 frames across the recording. Sampling randomly across each movie ensured that the median image included only the stationary background and not the animal. 1000-frame chunks were then loaded into memory and each frame was median-subtracted, down-sampled and Gaussian-blurred. The centroid of the brightest point after this procedure identified the approximate location of the centroid of the mouse, and a $400 \times 400$ pixel frame centered at these coordinates was kept in memory after mean subtraction. Finally the fully resolved clipped movie was saved as an h5 file for further analysis, along with an information file containing the coordinates used to clip the movie from the original frame. As a validation of comparability with past work, center of mass tracking [33] showed that in all experimental groups, mice covered the most distance on their first day of exposure to the open field, declining on later days [34, 35].

### Frame alignment and preprocessing

In total, 350 frames sampled from among clipped movies were used to train a deep neural network (DNN) using LEAP in order to identify the coordinates of the snout, center point and tail point (where the tail meets the body) of the mouse [21]. Each movie was then labeled using this network. The resulting body part coordinates were loaded along with each clipped movie and info file, and each frame of the original clipped movie was centered at the tail point and aligned to the tail point-to-nose axis using image translation and rotation functions in MATLAB. The centroid information was updated to incorporate the new centroid after translation, and the rotation values were also saved for each frame in an updated information .mat file.

### Body part coordinate identification

We used 660 frames to train a deep neural network (DNN) from the aligned clipped movies described in the previous methods [21]. The neural network was trained iteratively using the LEAP interface, where the network was used to label a sample set of frames that were then fixed manually and used as training data in order to update the network. In particular, the training set was designed to include examples of various postures during diverse behaviors that the network failed to label in early iterations. Frames with multiple occlusions of the head and limbs during grooming were trained with a ‘best guess’ of where the body part in question would be located, and the network was updated until all postures were labeled satisfactorily. Overall the network was trained to identify 18 body points (snout, ears, chin, inner and outer limbs, center, sides, tail point, tail center and tail tip), which would be used for behavioral classification.

### Classical measures using centroid tracking

Mouse centroid coordinates throughout each open-field recording were used to calculate basic measures of activity and spatial occupancy for each experimental trial. The centroid was used to calculate the velocity and position of the animal at any given time point, and this value was further used to calculate the spatial distribution of animals in the arena and a number of other metrics such as center crossings throughout all recordings.

### Statistics

All statistics were performed using MATLAB, R (rstatix, compositions, npmv, data.table, plyr, ggplot2, ggpubr, car, DescTools) or Python 2 and Python 3 (statsmodels, scipy, matplotlib, numpy). Data are presented as mean ± SD unless otherwise stated. Group mean comparisons were performed through two-way mixed analysis of the variance (ANOVA), repeated-measures ANOVA followed up by multiple comparisons post hoc tests using Bonferroni correction, unless otherwise stated or through one-way ANOVA followed by Tukey’s multiple comparisons test. For each comparison the effect size (Cohen’s d) was calculated. Normality was tested using the Shapiro–Wilk test, the equality (homogeneity) of variance across groups was tested using Levene’s test, homogeneity of the covariance matrices of independent samples was tested using Box’s M Test and the significance in variances of the differences was determined with Mauchly’s test of sphericity.

To investigate differences between the behaviors of mice in the open field, we performed analysis to account for the compositional nature of the data [36]. First, for each mouse, the set of fractions of time spent in each of the eight behavioral classes were transformed into isometric log ratio coordinates using the R package ‘compositions.’ In this coordinate space, differences between groups were analyzed using a nonparametric multivariate test (Wilks’ lambda-type test statistics) from the R package ‘npmv.’ To identify the behaviors that differ between groups, we calculated the bootstrapped 95% confidence intervals ($N=5000$) of the log ratio differences between different groups of mice for each behavior [36].

### Postural representations over time

Mouse bodies are not rigid and the choice of axis for egocentric alignment introduces bias that depends on the relative position of body parts used to establish alignment. Instead of aligning to a body axis, we used the distances between all pairs of virtual marker coordinates to capture the posture of the animal at each frame. We define the configuration of an animal as a set of two-dimensional coordinates, *x* and *y*, which describes the position of each tracked keypoint over each frame, *n*, given $i,j \in \{1: N_{keypoints}\}$. The distance matrix for a given frame $D_{n}$ was calculated as follows.

1 $$\begin{aligned} D_{n}(i,j) = \sqrt{((x_{n_{i}}-x_{n_{j}})^2 + (y_{n_{i}}-y_{n_{j}})^2} \end{aligned}$$

We used 11 high-confidence virtual markers to represent posture. The posture of an animal over a recording is now represented as an $11 \times 11 \times n$ array where *n* is the number of frames in a given movie. To simplify this matrix, we further remove all indices where $i \le j$, as these are always self-distances and equal to 0 (when $i=j$), or repetitions of the same distances (when $i<j$). We performed online principal component analysis (PCA) on the entire set of movies by sampling batches of frames and calculating the mean and covariance of *D* for each batch, keeping a running tab of the total mean and covariance across all movies and finally finding the eigenvectors of the ultimate covariance matrix. We projected the distance matrix onto the top 10 of these eigenvectors to produce a set of projections that captured about 90% of the variance in *D* with a greatly reduced dimensionality. The $10 \times n$ array describing the projections along the top ten principal components was saved for each movie and captured the posture of each animal over time.

### Temporal representation of behavior

We performed a wavelet decomposition on the projection matrix containing the top ten posture modes in order to find the power spectrum across a dyadically spaced set of frequencies between 0.25 and 20 Hz. The $10 \times n$ postural representation was transformed into a $(10 \times 25) \times n$ wavelet spectrogram where each of the ten postural modes was described by power along 25 frequencies. The relative power among different frequencies corresponds to how quickly the posture of the animal is changing in time, and power in high frequencies means that the projection along a particular postural mode is changing quickly. The postural dynamic representation described by the wavelets was the final signal used to compare time points from behavioral movies. We used the log of this signal, with all values below $-3$ set to $-3$ to cluster all recorded behavior into fine- and coarse-grained categories that were used in all further behavioral descriptions. The same transformation was applied to the wavelets from each movie during the reembedding step.

### Clustering and dimensionality reduction to form behavioral classes

Because of the unbalanced nature of our data, where extremely specific yet common behaviors like locomotion and a particular speed might overwhelm an automated clustering algorithm, we first used importance sampling to design a representative behavioral sample set, like we did with postures for training the DNN. We sampled movies from each experimental condition and created a dataset that encompassed all of the types of movements that were found in our recordings. This was particularly important for labeling uncommon behaviors that may not have appeared in many movies but were still behaviorally interesting.

In order to sample all behaviors present among our recordings, we first performed dimensionality reduction on the time series ${\mathbf {x}}_i$ from each movie independently using t-distributed stochastic neighbor embedding (tSNE) [37]. The resulting maps were sampled to find a variety of templates, or examples of the wavelet values, that were present in the given movie, ensuring that even rare templates were represented. These templates, on the order of 100 per movie used, were concatenated to form a training set that was then clustered into classes that formed the basis of our behavioral labels. We performed clustering using two methods that are complimentary when visualizing data: tSNE mapping and *k*-means clustering. First, as previously described [19], we performed dimensionality reduction on a training set of $\sim 40,000$ samples by embedding the 250-dimensional wavelet signal into a two-dimensional tSNE map. The final map can be used to cluster behaviors using the watershed transformation or to visualize the density of the behavioral repertoire used by an animal or group of animals. Instead of clustering in the two-dimensional map, we also performed *k*-means clustering on the training set, with a target of $k=100$ clusters. Comparing the tSNE and *k*-means methods reveals similar results; however, it is simpler to embed new samples into the *k*-means clusters by simply finding the nearest neighbor of a given sample and assigning it the cluster designation of the nearest neighbor.

### Posture dynamical fingerprinting

The power in the wavelet spectrum across tracked body parts can be used to interpret the results of behavioral clustering. For interpretability, the wavelet decomposition was recalculated using the raw displacement in real space for each tracked body part, in lieu of performing PCA to find a postural representation. The body part wavelet signals associated with time points assigned each to a given behavioral class were averaged and reshaped to generate a power spectrum. These ‘fingerprints’ detail the power in each frequency used in the decomposition for each tracked body part and correspond to visible features of the behaviors found in each cluster or class.

### Calculating behavioral time series and normalized usage of behavioral classes

Each 20-min behavioral movie contained approximately 96,000 frames sampled at 80 Hz. The posture dynamical clustering method we described previously was used to assign a behavioral label or class, from 1 to 100, to each of these frames. The creation of 100 clusters, more than were easily distinguishable by the human eye, allowed us to manually curate behavioral groups by viewing randomly sampled examples from each cluster. We then grouped behaviors into coarse groups that aligned with an understanding of behavior and the rodent phenotyping literature and also were already combined into building blocks based on our automated method.

Usage during a given experiment or segment of experiment was defined as the normalized histogram over behavioral classes. Evolution of behavior over the course of an experiment was found by choosing a time window, for example 180-s, and calculating this histogram over a sliding window of this size over the course of the experiment. The 95% confidence interval was calculated using the density values from each individual mouse.

### Finding locomotion bouts, steps and phase

Locomotion was first identified using the *k*-means clustering method. All time points that were identified as belonging to the locomotion class were found, and a structure of joint trajectories and centroid positions of bouts over 500 ms long was created for each movie. Each locomotion bout was then further characterized. First all bouts were up-sampled by a factor of 100 and *Z*-scored, and the findpeaks function in MATLAB was used to locate the time indices of the beginning of each stride for each of the four limbs. Peaks were required to be at least 80 ms apart and with a minimum peak prominence of 0.2 after *Z*-scoring. The peaks were used to assign a phase to each limb at each time point, where the end of the swing, or the start of upward motion of the paw, corresponds to $\theta =0$. For each time at which the front right paw has a phase of $\theta =0$, the phase of each other limb was sampled and recorded, along with the centroid speed and angular velocity of the animal, and the arena coordinates at which the step was performed.

## Results

### Deep phenotyping of open-field recordings

**Fig. 1:**
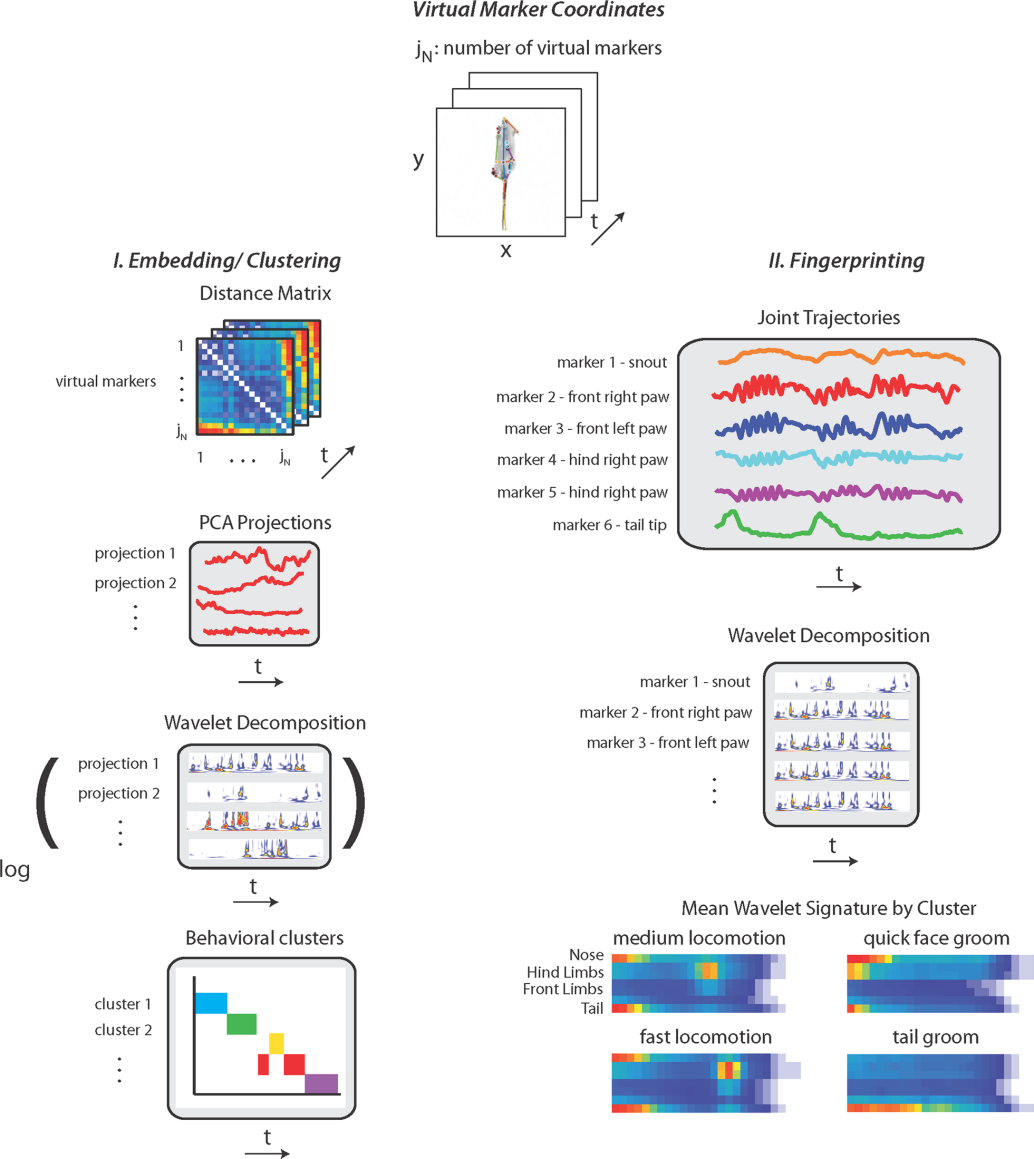
Processing of video recordings in the open field produces multi-scale quantitative descriptions of behavior. The pipeline takes virtual markers from pose estimation with LEAP to find behavior clusters and generate wavelet signatures. I. A visual representation of the embedding/clustering steps. Distance matrix calculated between virtual markers per each time frame transforms into the frequency domain and clustered using *k*-means. II. Raw joint trajectories are used to create wavelet signatures or ’behavioral fingerprints’ by finding the mean power spectrum during each behavioral cluster found in part I

We recorded over 700 trials in an open-field arena from 162 mice (Table 1). Each mouse was recorded for 20 min every day for a period of four days. We imaged the mice from below a transparent floor to allow observation of the paws and base of the tail. Movies were analyzed with a semi-supervised behavioral classification and behavioral labeling scheme (Fig. 1 and Additional file 1: Fig. S1). In the first step, we used a deep neural network to measure the posture of each animal over time. Recent advances in body part tracking allow for the use of a small set of user-generated labels to train a neural network for labeling body parts [21]. We trained the LEAP network with 660 human-annotated images and achieved position estimates with median confidence probabilities ranging from 0.91 to 0.98 for the snout, chin, inner and outer limbs, and base and tip of the tail. Ears, body center and sides, and the tail center point were excluded due to lower estimation accuracy (median confidence probability of 0.81). This pose estimation step resulted in a time series of the two-dimensional position of each body landmark, ${\mathbf {x}}_i(t)$. The next step in our analysis was a semi-supervised clustering of postural dynamics. Our previous work on behavioral clustering analyzed the dynamics observed in whole images of an animal but did not specifically probe the dynamics of individual body parts [19, 20, 22]. We adapted this method for use with the body part position time series, ${\mathbf {x}}_i(t)$ [21] and clustered the dynamics across timescales to define eight major classes of behavior in the open field (Fig. 1, Additional file 1: Fig. S1). The resulting ethogram revealed details not visible from centroid tracking alone (Additional file 1: Fig. S1a), namely the structure of spontaneous behavior across mesoscopic timescales.

**Table 1:** Summary of experimental strains and number of mice recorded

Strain	Number of mice
C57BL/6J Males	60
C57BL/6J Females	20
Cntnap2 knockout WT Littermates (male)	14
Cntnap2 knockout Heterozygote (male)	15
Cntnap2 knockout Homozygote (male)	10
L7-Tsc1 WT Littermates (male)	17
L7-Tsc1 Heterozygote (male)	17
L7-Tsc1 Homozygote (male)	9

Each mouse was recorded performing the open-field test on four consecutive days

The time series ${\mathbf {x}}_i(t)$ was rotated and translated into the reference frame of the animal by aligning with the anterior–posterior axis defined by the snout and tail base body parts and transformed into the frequency domain using wavelet decomposition. This produced a high-dimensional output that captured multi-scale (0.25–20 Hz) changes in posture over time (Additional file 1: Fig. S1c). We then performed *k*-means clustering of this high-dimensional data on a balanced selection of samples from recordings across all male mice. We initially clustered the sampled data into $k=100$ clusters, enough to achieve fine-grained clustering that in some cases was not distinguishable by eye. Data from all experiments were then clustered by assigning each time point to the cluster of its nearest neighbors in the *k*-means training set.

An exploration of all 100 clusters revealed several broader classes of behavior exhibited by mice in the open field (Table 2). Similar to recent findings from fruit flies [38], we found that mouse behavior could be organized into coarse classes with recognizable subclasses representing variable aspects of behavior. For example, the 21 clusters that made up the ’locomotion’ class represented the diversity of locomotion movements recorded, differing in velocity, amplitude and speed of limb movement, and the coordination of the limbs.

**Table 2:** Eight coarse behavioral classes are used to describe the 100 fine-grained clusters obtained from a *k*-means clustering of the posture dynamical signal

Class Label	Description
Idle	Mouse is still, no body parts are moving
Groom	Variety of movements that involve repetitive motions, mainly using the front limbs and head, but also including tail grooming and hind leg scratches
Slow Explore	Slow head turning and sniffing
Fast Explore	Quick or directed movements of the head, sniffing, some forelimb placements
Rear	Moving into reared posture, slow hover on hind limbs
Climb	Reared posture with limb movement occurring mainly at walls and corners
Amble and Turn	Slow or uncoordinated locomotion that does not appear regular, single steps
Locomotion	Regular directed movement that involves limb coordination

Descriptions were obtained by viewing movies sampling mice from each fine-grained behavioral cluster and grouping movies based on broad qualitative similarities

**Fig. 2:**
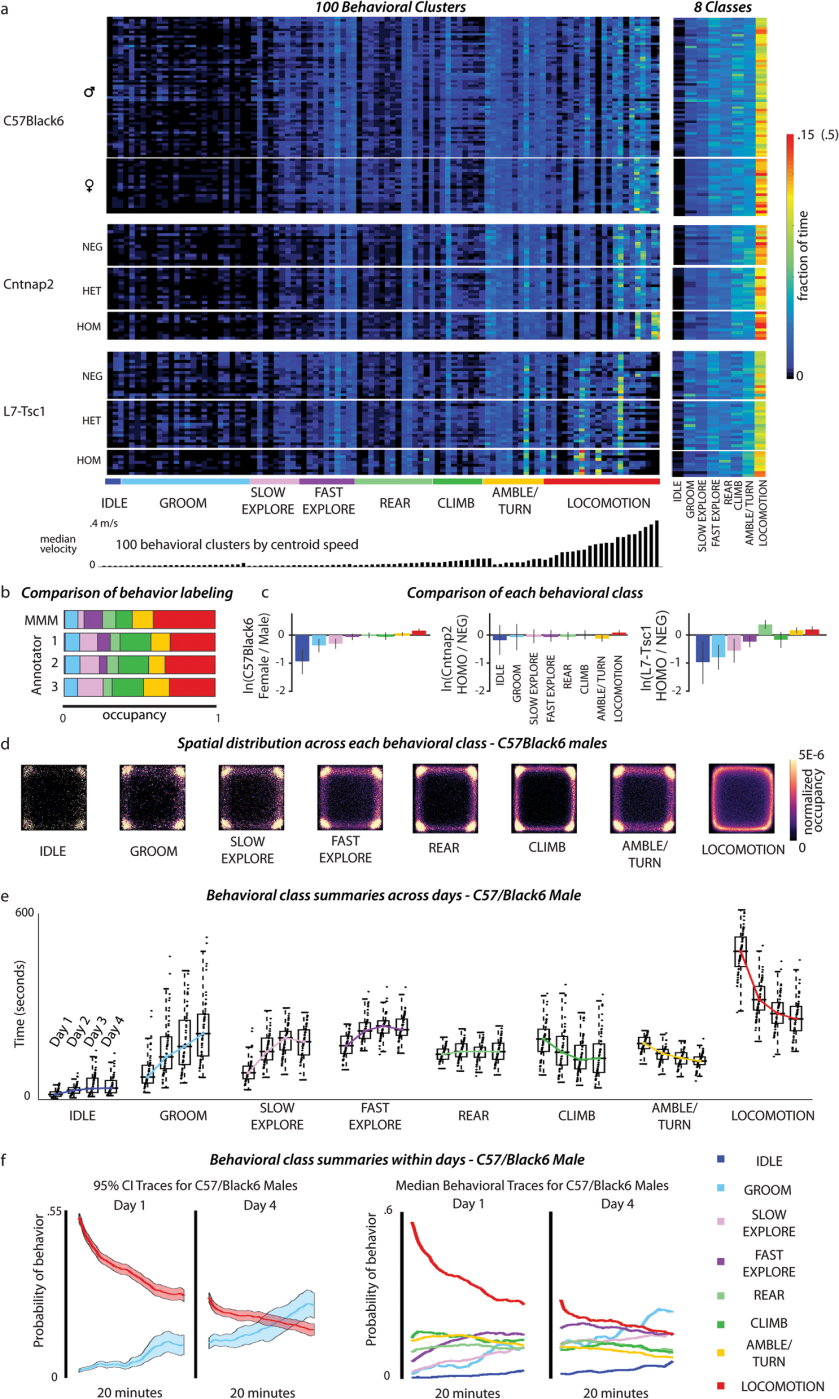
Spatial–temporal structure of C57BL/6J male mice behavior in the open field. **a** Behavioral cluster frequency across mouse models on Day 1. (left) Heatmap of the total behavioral occupancy of each behavioral cluster; rows are individual animals and columns are behavioral clusters. All rows within the 100-cluster matrix sum to one and describe behavior used over 20 min. Clusters are ordered by median centroid speed for each of the eight behavioral classes (bottom). (right) Heatmap of the behavioral occupancy for each of the eight behavioral classes. **b** Total occupancy of behavioral classes in a 20-min recording quantified by human annotators and by the algorithm. **c** Log ratio differences for all 8 behavioral classes between selected groups. The error bars show the bootstrapped 95% confidence interval (N = 5000, percentile bootstrap). **d** The spatial distribution is shown for each of the eight behavioral classes. **e** Behavioral usage for each of eight coarse categories plotted for C57BL/6J male mice for each of four observation days. All individuals are shown as points, colored traces correspond to the median fraction of time spent in the behavior for each day. **f** The usage of grooming and locomotion behaviors during the 20-min observation period for Days 1 and 4. Shaded regions represent the 95% confidence interval (left). The mean usage (right) for each of eight behavioral classes is shown for Days 1 and 4

We categorized each of the 100 clusters into one of eight behavioral classes (Additional file 1: Fig. S1 and Fig. 2a). This manual curation step revealed that time spent in the open field was composed of commonly studied behaviors such as locomotion and grooming, but also less distinct movements made for a large fraction of the time including spatial exploration and ambling. We defined the classes *fast exploration* and *slow exploration* to characterize periods that are usually unaccounted for in traditional measurement paradigms. Fast exploration, for example, included quick turns and sniffs that are characteristic of alertness or anxiety, whereas slow exploration included head sweeps and extensions during calm periods.

We validated the accuracy and interpretability of these classes by visually inspecting randomly sampled movies of behavior from each of the 100 clusters and the eight classes. Movie segments were sampled from all C57BL/6J male movies (300 20-min movies overall) and limited to examples where the behavior in question was performed for at least 250 ms or 20 frames at our frame rate of 80 Hz. Examples from the 100 behavioral clusters and eight behavioral classes are available in the Supplementary Materials. We also compared the eight classes with manual annotations by three researchers who were instructed to label each frame after reading the descriptions from Table 2 and watching selected video snippets. Annotators showed high agreement with one another, but annotators and the algorithm agreed less often (Additional file 3: Fig. S3a, c). Human-vs-algorithm mismatches may have arisen from the difficulty of conceptually capturing a class with a category name. In addition, the algorithm uses spectrograms whose power is concentrated at fast timescales, whereas human annotators may use longer timescale information. This possibility is supported by the fact that the algorithm captured the beginning and end of locomotion bouts to match the limb kinematics, whereas human annotators would often classify beginnings and ends into other behaviors. Human annotators also tended to merge bouts of behavior into a single category (Additional file 3: Fig. S3b); for example, human annotators might combine a series of ‘rear’ and ‘climbing’ episodes as a single bout of ‘climbing’ where the algorithm made subdivisions based on high-movement hindlimb lifts from the arena floor. Altogether, differences between manual annotations and semi-supervised behavioral classifications were reflected in the fractional occupancy by each behavior (Fig. 2b).

### Behavioral differences upon introduction to the open-field arena

The fraction of time spent in each cluster in the experimental arena on the first day of exposure (day 1) is shown in Fig. 2. Clusters in Fig. 2a are ordered horizontally according to their behavioral class and then by median centroid speed within each class. Mice spent the most amount of time on day 1 locomoting after placement in the novel arena, followed by turning, climbing and rearing, but almost no time in the idle state.

Inspection of the occupancy of locomotion modes across individuals revealed features that set the Cntnap2 KO and L7-Tsc1 mutants apart from their WT littermates, as well as from the large number of C57BL/6J mice, which had the same genetic background. C57BL/6J mice were most likely to use the fourth- and fifth-fastest locomotion modes at around 0.2 m/s on Day 1. The Cntnap2 KO was distinct from WT and heterozygote littermates by the enriched usage of the several fastest locomotion clusters and a decrease in more moderate locomotion (Fig. 2a). This result was in agreement with findings that Cntnap2 KO mice are hyperactive [27] and accounted for the large distance these mice covered on the first day in the open field (Additional file 2: Fig. S2). Full mutants from the L7-Tsc1 group showed the opposite trend from Cntnap2 KO mice, spending less time in the fast locomotion clusters than any other group. The L7-Tsc1 mutants often used several clusters of slow locomotion that were uncommon in either their control littermates or the baseline C57BL/6J mice.

To investigate overall differences in the time spent in the eight behavioral classes, we performed a compositional data analysis [36]. After transformation of the fractions into isometric log ratio coordinates, differences between groups were analyzed using a nonparametric multivariate test (Wilks’ lambda-type test statistics). For day 1, we found significant differences at $\alpha = 0.05$ between female and male C57BL/6J mice. The behavior of male and female C57BL/6J mice was significantly different from Cntnap2 KO mice and their littermates as well as L7-Tsc1 mutants and their littermates, except for between female C57BL/6J and Cntnap2 KO mice. L7-Tsc1 mutants behaved differently than their littermates and Cntnap2 KO mice. No significant difference in the time spent overall the eight behavioral classes between Cntnap2 KO mice and their littermates was observed. To gain further insights into the behaviors that caused the differences between the groups, we calculated log ratio differences between groups for each behavior (Fig. 2c) [36]. Female C57BL/6J mice spent reduced time in slower behaviors (idle, groom, slow explore) and an increased time in locomotion compared to male C57BL/6J mice. A similar observation was made when comparing L7-Tsc1 mutants with their WT littermates on day 1: they spent less time being idle, grooming or slow exploring, but more time rearing, turning and in locomotion.

### Spatial habituation is reduced in L7-Tsc1 mutant mice

Mice tend to avoid open spaces in natural environments in an attempt to avoid predators. Traditional analyses of open-field experiments divide the arena into zones to quantify time spent in the open space, near the edges and in the highly confining corners. Mice spend most of their time at the edges and corners (thigmotaxis). Previous researchers have sometimes interpreted thigmotaxis in terms of the emotional state of the mouse, often anxiety [39]. We found that in these corner positions, mice performed a variety of behaviors including grooming, rearing, climbing and exploration (Fig. 2d).

When first introduced to a new environment, mice explored the full space with some preference for corners. With time and repeated exposure to the same environment, mice habituated and spent less time crossing through the center of the arena and more time in the corners (Additional file 2: Fig. S2c, d). We measured the time an animal spent in the corner zones of the arena for each of the 4 days of observation to identify the development of thigmotaxis. C57BL/6J males, as well as WT and heterozygote littermates of L7-Tsc1 mutants and Cntnap2 KO mice, demonstrated a gradual increase over days in time spent in the corners (*d* range from 0.87 to 2.11 for day 1 compared to day 4, repeated-measures ANOVA for each group, $p < 0.01$), while C57BL/6J females, L7-Tsc1 mutants and Cntnap2 KO mice all failed to show a significant increase of time spent in the corners (repeated-measures ANOVA, $p = 0.13$, $p = 0.28$, $p = 0.58$) (Additional file 2: Fig. S2c). Compared to males, female C57BL/6J mice spent on average 15% less time in the corners on day 1 (d = 0.83, one-way ANOVA, groups-by-day 1, $F(7,154) = 8.83$, post hoc $p = 0.008$) and 26% on day 4 (d = 1.43, groups-by-day 4, $F(7,154) = 11.15$, post hoc $p = 0.0002$). In summary, a propensity toward corners after multiple days was sex-dependent in C57BL/6J mice and failed to occur in L7-Tsc1 mutant and Cntnap2 KO mice.

Reduced spatial habituation was also seen in the number of center crossings for L7-Tsc1 mutants (repeated-measures ANOVA $F(3,24) = 4.24$, $p = 0.12$, $F(3,48) = 47.76$ and $F(3,48) = 34.36$ for littermates $p < 0.001$). L7-Tsc1 mutant mice showed similar initial values of center crossing as WT littermates on day 1 (one-way ANOVA, p = 0.39). But after four days of habituation, mutants showed a tendency to cross to and from the center zone more frequently than WT littermates (one-way ANOVA, groups-by-day 4, post hoc $p = 0.07$) (Additional file 2: Fig. S2d).

### Behavioral habituation is reduced in L7-Tsc1 mutant mice

Mice also modulate specific behaviors as they habituate to a new environment. All experimental groups traveled significantly less on the second day compared to the first day ($d = 1.5, 2.1, 2.8, 1.8$ for female, male C57BL/6J mice, Cntnap2 KO and L7-Tsc1 mutant mice, repeated-measures ANOVA for each group, $p < 0.05$) (Additional file 2: Fig. S2b). We found that both female and male C57BL/6J mice increased the time they spent idling, grooming, exploring and rearing on day 5 over day 1 ($d = 0.9, 1.2, 1.6, 0.5$ for females and $d = 0.9, 1.6, 1.6, 0.7$ for males; repeated-measures ANOVA for each group, $p < 0.01$) and decreased the time they spent locomoting ($d = 1.9$ for females and $d = 3.2$ for males, $p < 0.01$) in the same arena. The largest day-on-day shift occurred between the first and second day in both female and male C57BL/6J mice (Fig. 2e and Additional file 4: Fig. S4a). Male C57BL/6J mice in addition spent significantly less time climbing and turning ($d = 0.5$, repeated-measures ANOVA, post hoc $p < 0.01$ and $d = 2.0$, post hoc $p < 0.01$) in contrast to female C57BL/6J mice (repeated-measures ANOVA, $F(4,72) = 1.36$, $p = 0.51$ and $F(4,72) = 2.47$, $p = 0.17$).

Behavior also changed within the 20-min observation period of each experiment (Fig. 2f). The pattern of behavioral change exhibited by C57BL/6J mice within an observation period was similar to the across-day change: locomotion, climbing and turning decreased over time while idling, exploring and grooming increased (Fig. 2e). A comparison of within-day habituation curves for each of eight behavioral classes over five days of recording revealed this trend across all behaviors, with females performing active behaviors more than males for the entire duration of each experiment (Additional file 4: Fig. S4).

**Fig. 3:**
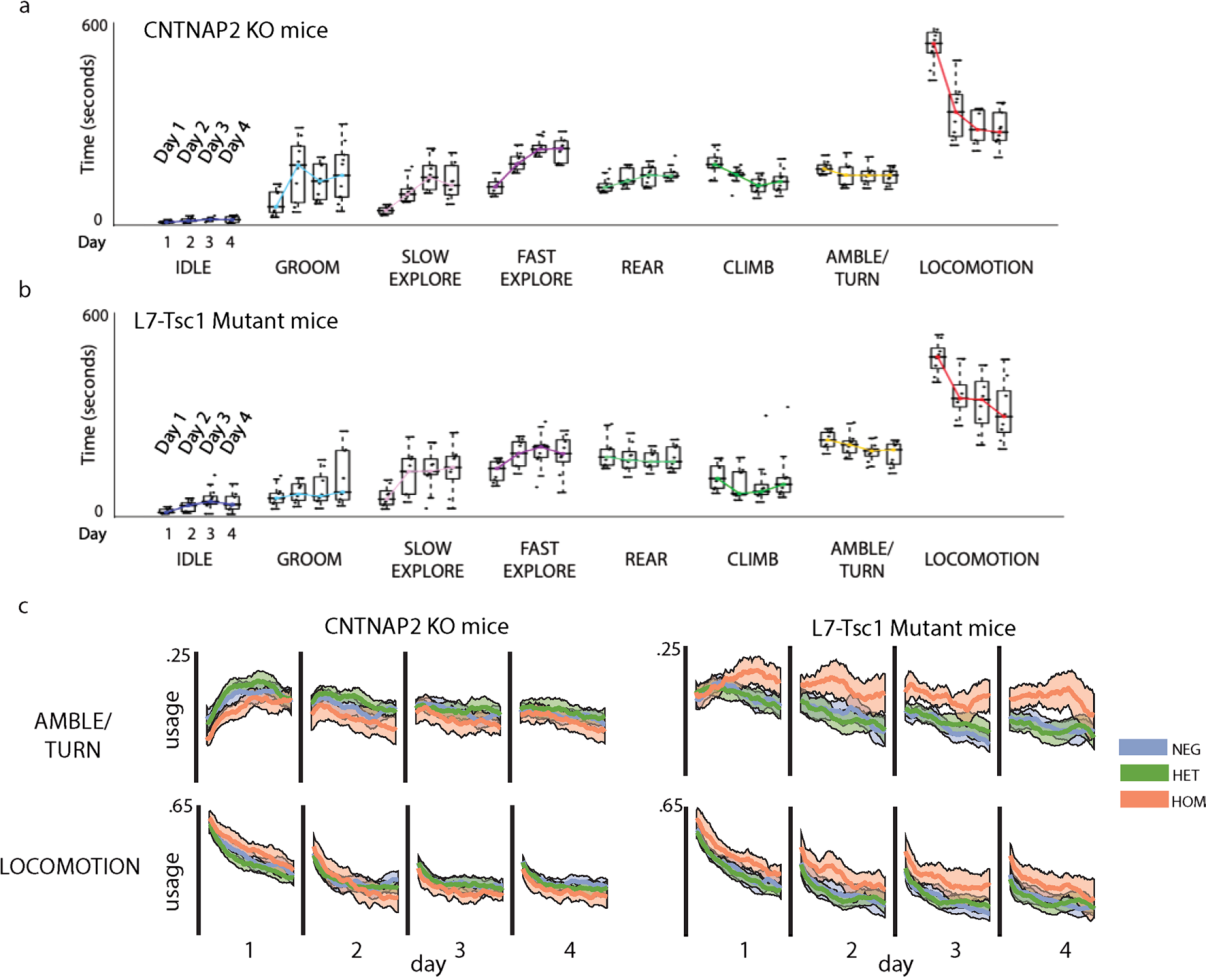
Behavioral usage over time for Cntnap2 KO and L7-Tsc1 mutant mice. **a**, **b** Behavioral usage for each of eight coarse categories plotted for **a** Cntnap2 KO and **b** L7-Tsc1 mutant mice for each of four observation days. All individuals are shown as points, colored traces correspond to the median fraction of time spent in the behavior for each day. **c** The usage of turning and locomotion behaviors during the 20-min observation period for Days 1 through 4 for Cntnap2 knockouts and littermates (left) and L7-Tsc1 mutants and littermates (right). Colors indicate the strain (blue—WT, green—heterozygote, orange—homozygote). Shaded regions represent the 95% confidence interval

Cntnap2 KO mice were unaltered in their ability to habituate within-day and their behavior across days was not significantly different from their littermates (two-way mixed ANOVA idle: $F(2,36) = 2.96$, $p = 0.07$; slow explore: $F(2,36) = 1.92$, $p = 0.16$; fast explore: $F(2,36) = 1.56$, $p = 0.22$; rear: $F(2,36) = 0.09$, $p = 0.92$; climb: $F(2,36) = 2.24$, $p = 0.12$), although they did groom significantly more often on day 2 compared to WT littermates (one-way ANOVA, $F(2,36) = 6.07$, post hoc $p = 0.02$) (Fig. 3 and Additional file 5: Fig. S5). In contrast to Cntnap2 KO mice, L7-Tsc1 mutant mice showed reduced within-day and across-day habituation (Fig. 3 and Additional file 6: Fig. S6).

**Fig. 4:**
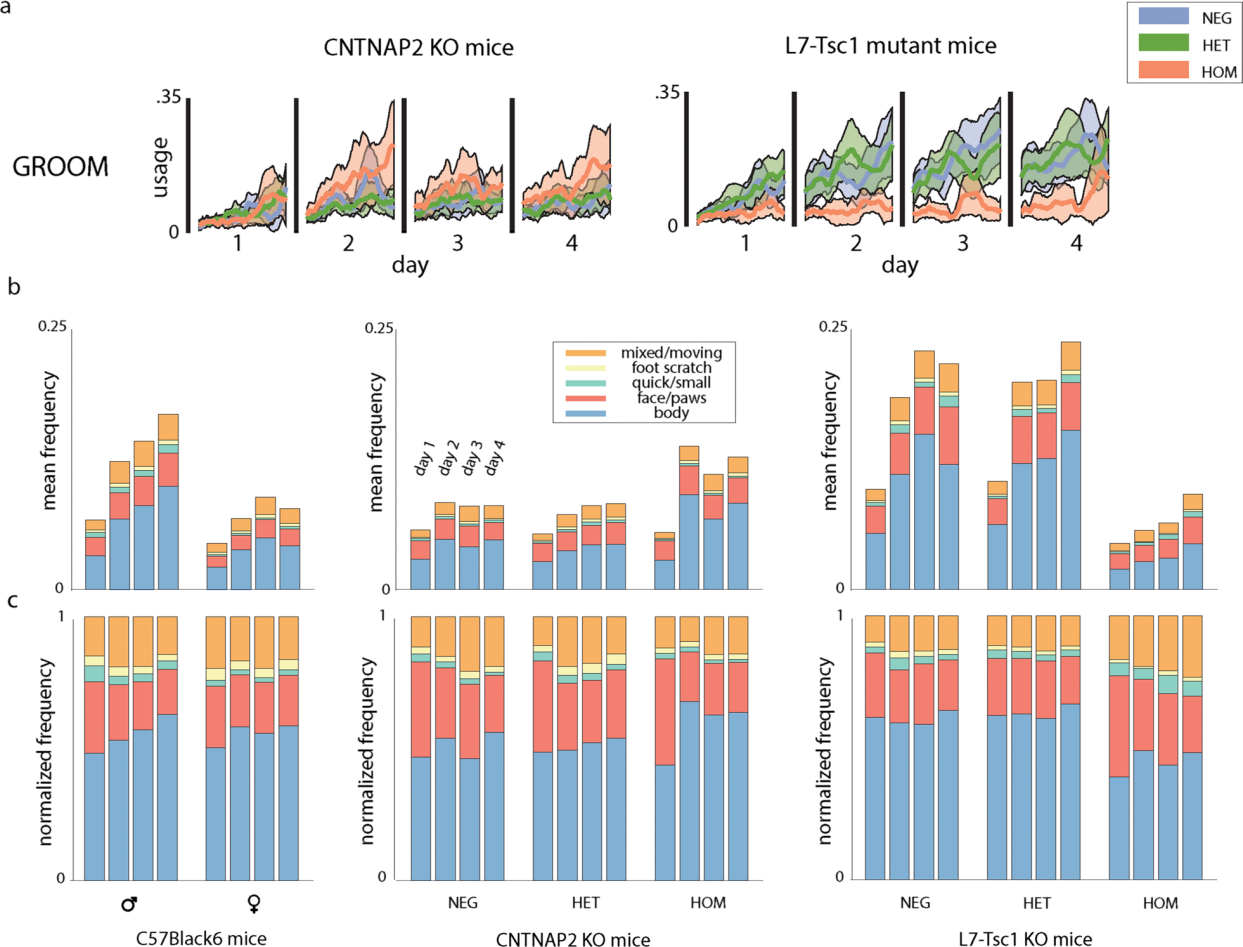
Habituation of grooming behaviors **a** The usage of grooming behaviors during the 20-min observation period for Days 1 through 4 for Cntnap2 and L7-Tsc1 mice. Colors indicate the strain (blue—WT, green—heterozygote, orange—homozygote). Shaded regions represent the 95% confidence interval. **b** Stacked bar plots showing the mean frequency across mice for each of five grooming behaviors across Days 1 through 4 with corresponding stacked bar plots below showing the normalized mean frequency for each of five grooming behaviors (**c**)

The defect in L7-Tsc1 mutant habituation was most apparent in locomotion and turning behaviors (Fig. 3 and Additional file 6: Fig. S6). These mice did not show the expected reduction in turning either over days or within the 20-min observation period. Turning was used more often on day 1 (one-way ANOVA, $F(2,40) = 5.62$, post hoc $p = 0.03$) and did not decrease to the same degree over the observation time as WT and heterozygote littermates. Locomotion decreased over time but to a lesser degree and the level of locomotion in the L7-Tsc1 mutant mice was significantly higher than in WT littermates for days 1, 2 and 3 (one-way ANOVAs, groups-by-day, all post hoc $p < 0.05$).

### Grooming behaviors vary in a complex manner in neurodevelopmental mouse models

Grooming refers to a variety of repetitive self-touching behaviors, and mouse self-grooming has been used as an animal model for the self-stimulating behaviors observed in autism [40]. Previous reports have limited quantification to 10 min of observation and have not distinguished different types of self-grooming or reported sex differences [29, 41, 42]. In our four-day recording period, we observed that all mouse groups groomed more frequently as the days passed (Figs. 2e, 3a, b, 4, Additional file 4: Fig. S4a, Additional file 5: Fig. S5a and Additional file 6: Fig. S6a), with larger increases in males compared with females (males $d = 1.6$ and females $d = 0.6$ day 1 compared to day 4, repeated-measures ANOVA, $F(4,236) = 32.61$ and $F(4,72) = 9.60$, post hoc $p < 0.01$). We furthermore found that within-model contrasts (male v. female, Cntnap2 KO v. Cntnap2 WT, L7-Tsc1 mutant v. L7-Tsc1 WT) were modest on day 1 but grew considerably over the next three days. The two neurodevelopmental mouse models showed opposite trends: Cntnap2 KO mice starting from day 2 groomed more frequently than their littermates (one-way ANOVA day 1: $F(2,36) = 0.07$, $p = 0.93$; day 2: $F(2,36) = 6.07$, $p = 0.02$), whereas L7-Tsc1 mutants mice groomed much less frequently than littermates cross all days (one-way ANOVA day 1: $F(2,40) = 5.28$, $p = 0.04$; day 4: $F(2,40) = 5.27$, $p = 0.04$) (Fig. 4).

The grooming class could be further divided into several modes of grooming: body grooming, face/paw grooming/licking, small quick movements, foot scratching and a mixture mode to account for time spent readjusting between modes. To validate grooming modes, human annotators were presented with movie segments that included grooming based on the algorithm and then were asked to annotate videos frame by frame (Additional file 3: Fig. S3d). A comparison indicated that the algorithm emphasized shorter timescales and identified more mixed bouts of grooming than human annotators (Additional file 3: Fig. S3e). The total number of episodes spent grooming was normalized to 1 to obtain a relative usage of these grooming modes (Fig. 4b). Despite differences in total grooming frequency between C57BL/6J males and females, their relative grooming-mode frequencies were similar and showed the same trend over time. Across days, body grooming increased in relative frequency (repeated-measures ANOVA, $F(1,77) = 8.62$, $p = 0.02$) while face grooming and quick grooming movements became less frequent (repeated-measures ANOVA, $F(1,77) = 8.74$, $p = 0.02$ and $F(1,77) = 6.96$, $p = 0.048$).

Both neurodevelopmental mouse models showed altered distributions of the grooming behaviors. Cntnap2 KO mice exhibited more body grooming compared to their WT littermates on day 2 (one-way ANOVA $F(2,36) = 7.11$, post hoc $p = 0.02$). L7-Tsc1 mutant mice showed the opposite effect with less body grooming on all days of observation (one-way ANOVA, $F(2,40) = 5.53$, each day, post hoc $p < 0.05$) and more face/paw grooming on days 3 (one-way ANOVA $F(2,40) = 7.59$, post hoc $p = 0.002$) than WT littermates (Fig. 4c).

In all groups except for L7-Tsc1 mutants, grooming frequency also increased dramatically within each day’s observation period. Cntnap2 KO mice and their littermates groomed with similar frequency at the start of each day’s observation, but knockouts increased the time spent in grooming at a greater rate during each 20-min period (Fig. 4a). L7-Tsc1 WT and heterozygote mice likewise groomed more within each day’s observation period, but L7-Tsc1 mutant mice did not. These measurements show that systematic patterns of variation in grooming within each day of observation typically exceeded the variation across days.

### L7-Tsc1 mutant and Cntnap2 KO mice show travel differences and gait defects

**Fig. 5:**
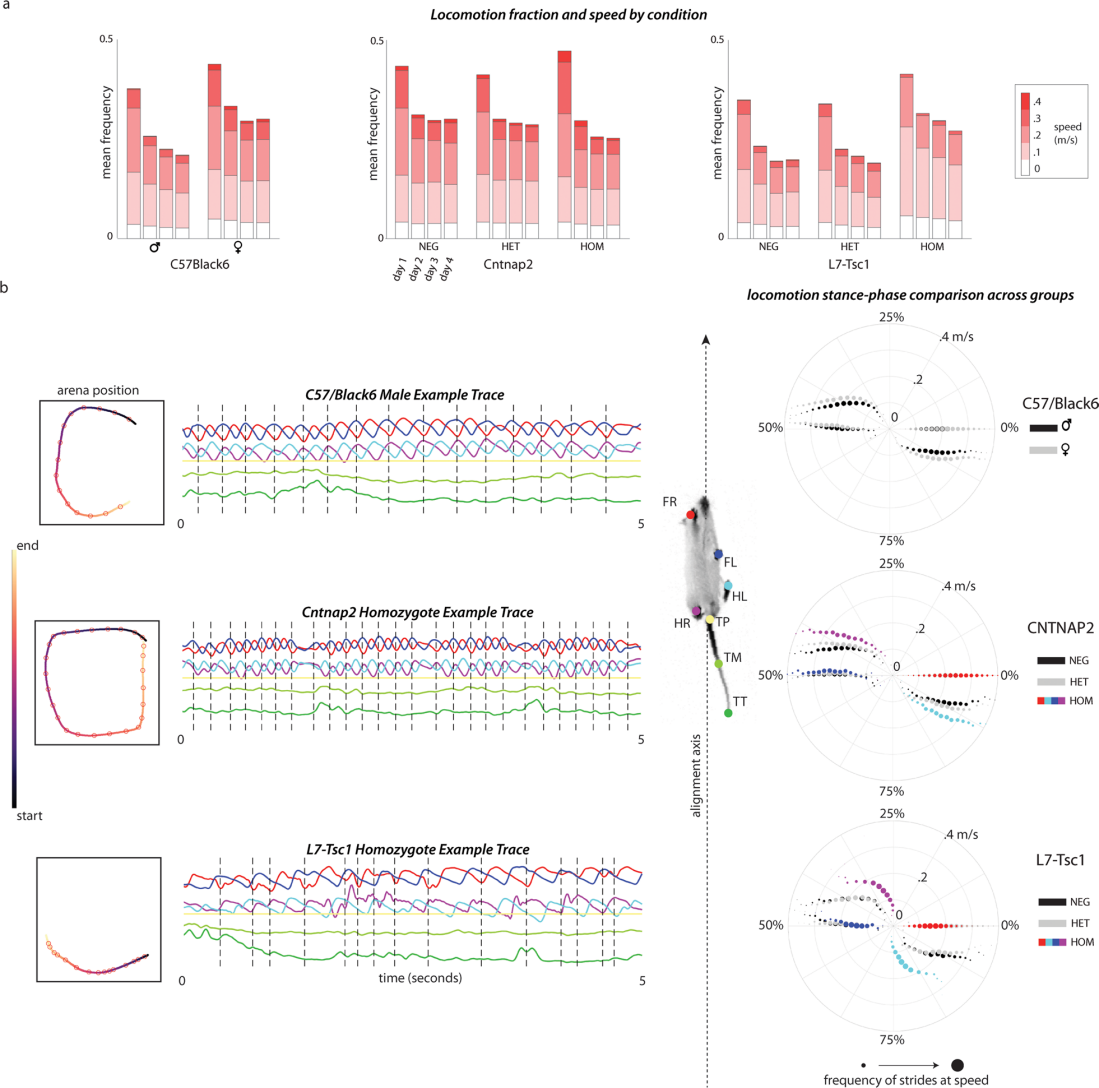
Locomotion kinematics are altered in the neurodevelopmental mouse models. **a** Stacked bar plots showing the mean frequency for each of four speed bins on Days 1 through 4. **b** Left: Examples of the motion of the limb and tail points during 5-s bouts of locomotion for C57BL/6J, Cntnap2 KO and L7-Tsc1 mutants. The trajectory of the centroid of the mouse is color coded by time. Time series of the position of each body part projected onto the anterior–posterior axis is plotted. The start of each gait cycle is marked with a dashed black line and defined using the front right paw (FR, red). Center: An example image of a mouse is labeled with colors used for each body part, and the axis of body alignment used when segmenting strides is shown as the axis between the nose and tail base point (TP). Right: Polar plots of the phase $\theta$ of the gait cycle in which each paw reaches a minimum along the alignment axis in the body frame. The front right paw is used to define $\theta =0$. The radial axis indicates the speed of locomotion, which was used to bin the phase results, and the size of the circles indicate how frequent that particular speed was for a given condition. For Cntnap2 KO and L7-Tsc1 mutants, the colors of dots correspond to the four labeled paw points

All experimental conditions showed reduced amounts of locomotion after day 1. However, the distribution of locomotion speeds depended on condition. In C57BL/6J mice, female mice moved on average 11% faster ($d = 1.21$, one-way ANOVA $F(7,154) = 33.47$, $p < 0.001$) than their male counterparts. Female C57BL/6J mice more often locomoted at speeds above 0.3 m/s than males (Fig. 5a). Cntnap2 KO mice did not differ from their littermates in the median velocity ($p = 0.11$) or distance traveled ($p = 0.12$) (Additional file 2: Fig. S2), although they did locomote at speeds above 0.3 m/s significantly more often on day 1 (one-way ANOVA, $F(2,36) = 11.58$, post hoc $p < 0.01$). In contrast to all other experimental groups, L7-Tsc1 mutant mice kept their median velocity across days (repeated-measures ANOVA $F(3,24) = 1.26$, $p = 0.31$) compared to WT littermates ($F(3,48) = 9.46$, $p < 0.001$). The distance that L7-Tsc1 mutants traveled on days 1 to 4 was not significantly different from their WT littermates (one-way ANOVA, group-by-days, post hoc $p = 0.60$, $p = 0.33$, $p = 0.66$, $p = 0.13$). They did so by moving more often (one-way ANOVA, group-by-day 1, post hoc $p = 0.02$) but at 19% slower median velocity ($d = 1.55$, one-way ANOVA, group-by-day 1, post hoc $p = 0.001$) (Fig. 2c and Additional file 2: Fig. S2a): L7-Tsc1 mutant mice significantly increased the number of locomotion bouts at 0 - 0.2 m/s (one-way ANOVA, group-by-day, each day post hoc $p < 0.001$), a difference that persisted across all four days of testing (Fig. 5a).

Nearly all children with autism show motor impairment [43, 44], with gait following a locomotor pattern resembling cerebellar ataxia [45, 46]. To explore the kinematics of gait, we identified all locomotion bouts (Figs. 1g, 5b) and analyzed individual body part trajectories along the anterior–posterior axis. As expected, the front and hind paws moved in an oscillating pattern in the frame of reference of the mouse (Fig. 5b) [47]. We also found that the tail often oscillated as well, matching the frequency of the paw oscillation.

Mouse locomotion could be broken into two different gaits depending on the speed. At very slow speeds ($v<0.1$ m/s), C57BL/6J mice used a *walking* gait with paws moving in sequence, each for approximately one quarter of the walking cycle (Fig. 5b). The order of movement and coordination in the walking gait was Front Right, Hind Left, Front Left, Hind Right. At higher speeds, mice transitioned to a *trot* gait with alternating diagonal pairs of legs moving together. Left–right pairs of legs were not in phase with each other within the gait cycle. We found that for speeds above $\sim 0.1$ m/s, C57BL/6J mice had an average phase difference of $\sim 0.5$ rad or about 8% of the gait cycle. This phase offset decreased with speed from about $\sim 1$ rad at $v=0.1$ m/s to $\sim 0.25$ rad at $v=0.4$ m/s.

The two types of neurodevelopmental mutants displayed differences in both the walk-to-trot transition speed and the phase difference between opposite-limb (FL/HR and FR/HL pairs) movements. Cntnap2 KO mice showed a similar transition speed of about 0.1 m/s as their littermates. In contrast, L7-Tsc1 mutants used the walking gait much more and transitioned to the trotting gait at a much higher velocity of $\sim 0.2$ m/s. The phase difference during trotting was larger for both neurodevelopmental mouse models. Cntnap2 KO had a phase difference at 0.2 m/s of 0.7 rad compared to a difference of 0.5 rad for their littermates. L7-Tsc1 mutants had an even larger difference of 1.0 rad at 0.2 m/s compared to 0.5 rad for their littermates.

## Discussion

We have found that the observation of mice in an open arena is sufficient to characterize behavior on scales ranging from sub-second individual limb movements to multi-day adaptive change. The two neurodevelopmental mouse models we used, Cntnap2 KO and L7-Tsc1 mutants, differed in distinct ways from WT mice on within-day measures of behavior, including gait defects and self-grooming. In addition, L7-Tsc1 mutant mice showed defects in multi-day behavioral evolution. Thus our methods can quantify complex behavior and differentiate between mouse models’ capacity to express these phenotypes (Additional file 7: Fig. S7).

Animal behavior is high-dimensional. Changes in movement, transitions between states and adaptive change are most easily observed by different metrics on multiple timescales. Our method for semi-supervised behavioral classification allows exploration of all three types of dynamics from a single video recording. Such deep phenotyping can capture rich behavioral readouts that illuminate the effects of genetic and experimental perturbations in naturalistic environments.

Previous methods for analyzing freely moving behavior have been limited to restricted feature sets. Observations from freely moving animals have focused on simple measures, such as spatial location, using footprint measurement and centroid tracking. Detailed limb movements have been observed in constrained environments such as a corridor or treadmill to model the relationship between gait and postural features [47, 48], but this approach does not capture movement in a more natural context. Our approach allows all these types of measurements to be made at once in an automated manner.

The simplicity of the open field allows rapid characterization without need to train mouse or experimenter. This level of automation and standardization may contribute to differences with past work. For example, we found that L7-Tsc1 mutant mice showed reduced grooming compared with wild types, yet past studies report increased grooming. We found that grooming included many sub-states that involved different movements. Differences in how human scorers classify such states may lead to laboratory-to-laboratory variability in quantification. Additional sources of variation include conditions that facilitate grooming, such as spraying mice with water or including bedding material in the chamber. Conversely, our automated methods incorporated a temporal window for clustering, so that singular quick grooming movements might have been missed. Further study is needed to reconcile these approaches.

Previous studies of mouse behavior have identified large sex differences in pairwise interactions (i.e., same-sex vs. opposite-sex interactions). Our analysis identified robust sex differences even in the absence of a specific task or another mouse in the chamber. The male-versus-female difference was also seen in Cntnap2 KO-versus-WT mice, where knockouts tended to display increased activity. In contrast, L7-Tsc1 mutant mice showed a reversed phenotype from the Cntnap2 KO mice and presented with slower and less coordinated behaviors. Our findings recall the observation that men are identified as being on the autism spectrum four times as often as women, and would be consistent with the possibility that regulation of some genes (by our measures, *Cntnap*2) but not others (*Tsc*1) might have differential effects by sex [49].

Mouse models of autism are typically generated from a focal perturbation affecting as little as one gene. However, a single-gene disruption can still affect many cell types and brain regions at once. Cntnap2 is expressed in neocortex, striatum, hippocampus and cerebellum [27]. Therefore behavioral changes in Cntnap2 KO mice may arise from perturbation of any of these regions or a combination of them. In contrast, L7-Tsc1 mutant mice exhibit reduced *Tsc*1 expression specifically in Purkinje cells, thus influencing behavior through cerebellar impacts on brain activity. The patterns of disruption are therefore likely to differ. For example, Cntnap2 KO mice show reductions in neocortical spine number [50] whereas L7-Tsc1 mutant mice show increased neocortical dendritic spine density [51].

The ability to simultaneously characterize movement kinematics and larger-scale features of behavior, even without a task condition, has particular relevance to the study of autism. Movement disruptions appear in 80% of children with autism [43, 44], despite the fact that movement is not considered part of the classic triad of defining symptoms. Injury to the cerebellum at birth leads to a sharply increased likelihood of autism, raising the possibility that the same structure that regulates smooth movement may also play a key role in driving the development of higher-level behavioral capacity [52]). In this way, movement and cognitive maturation may use shared neural substrates. Our analysis of Cntnap2 KO and L7-Tsc1 mutant mice reveals a shared disruption to specific parameters of gait arising from different genetic perturbations, one brain-wide and one cerebellar. We find that each model exemplifies different features: hyperactivity in one case, and failure to adapt in the other. In the future it may be possible to use alterations in movement to aid in the classification of autistic individuals. In addition, early life identification of motor symptoms may allow rapid risk stratification for early intervention.

Mouse tests for autism-like phenotypes were selected for their putative relevance to human behavior and for their ease of administration. However, it is often difficult to probe system-level mechanisms responsible for the observed alterations in performance. For example, Cntnap2 KO mice [27, 28] show disruptions on a wide array of tests, none of which are well studied at a circuit level. Deep behavioral phenotyping allows system-specific defects (gait) and higher-order performance to be assayed within the same behavioral recording. In this way our methods open the future possibility of investigating complex behavior with substantially increased depth, both of phenotyping and of neural mechanism. The power of these methods will increase as they are integrated with specific task or test conditions, as well as neural recording and perturbation.

## Limitations

ASD patients show sex-dependent differences in behavior and neurobiology; we used wild-type mice of both sexes and male ASD-model mice for experiments. Therefore, conclusions driven from our results might not reflect behavioral alterations in the female ASD-model mice. Approximately a dozen mouse models of the genetic risk factors for ASD have been developed, of which we studied only two. The pipeline, which provides a solution that automatically classifies complex behaviors in large datasets, can be applied across available strains to facilitate systematic studies of behavioral mechanisms, determine heritable components and potentially link mouse phenotypes to human psychiatric symptoms. This method also enables longitudinal and rescue studies of mouse behavioral dynamics. Finally, we did not track the social behavior. A recent implementation of SLEAP [53] allows multiple-animal pose tracking and enables future studies of posture dynamics in the social context. Finally, this study was done without any task condition and does not explore complex interactions between a mouse and its environment or with other mice. Such studies are needed to further compare and contrast mouse and human behavior.

## Conclusions

Our approach to characterizing behavior, which enables fine spatiotemporal disambiguation of behaviors enriched in animal models of ASD, may also be useful in the understanding of human autism and other neurodevelopmental disorders. Predictors of autism such as unusual patterns of sensory response are known to emerge in the first year of life. Deep behavioral phenotyping can expand the range of observations without need for a specific task. These methods may also be extended to include social interactions using methods for multi-individual tracking such as SLEAP [53]. By characterizing fine-grained movement, limb coordination and long-timescale adaptation, it may be possible to identify distinctive features of behavior that precede a formal diagnosis of ASD. Such information can potentially stratify at-risk children according to different points of departure from neurotypical paths of cognitive and social development. As the brain-wide mechanisms of autism and other neurodevelopmental disorders become better understood, such stratification may aid in the better targeting of treatment.

## Supplementary information

**Additional file 1: Fig. S1.** **a** Ethogram of behavioral classes produced from our behavioral clustering and classification pipeline based on the posture and movement of animals over time. Below, the same time series is visualized as a raster to demonstrate behavioral usage during a 2-min period. **b** Mouse centroid speed over the same 2-min period shown in panel (**a**). **c** Raw power for select body parts. N: nose, FR: front right foot, FL: front left foot, HR: hind right foot, HL: hind left foot, TT: tail tip. **d** Position time series for the nose, front and hind feet, and tail tip (left) during two behaviors. The *y* position, the anterior–posterior axis, is shown for a locomotion bout (center). The *x* position, the medial–lateral axis, is shown for a grooming bout (right). We use the full 2D position of each body part in our analyses, but show only the dominant axis for these behaviors here for brevity. **e** Normalized power spectra for several tracked body parts for each of the eight behavior classes. **f** Visualizations of the tSNE embedding for a subselection of data show how the methods are related.

**Additional file 2: Fig. S2.** Common metrics for open-field performance plotted for all conditions. **a** Median velocity during locomotion, **b** Median distance covered during 20 min in the open field. **c** The total time spent in the corner regions for each mouse on each day displayed as a box plot. The median value is shown as a solid line in the color corresponding to the given condition. **d** The number of center crossings for each mouse on each day displayed as a box plot. The median value is shown as a solid line in the color corresponding to the given condition.

**Additional file 3: Fig. S3.** Annotation of mouse behavioral classes. **a** Ethogram of behavioral classes produced from MouseMotionMapper (MMM) and individual annotators. **b** Typical agreements and discrepancies between MMM and human annotators synchronized with body parts trajectories. **c** Quantification of the agreement overlap between individual annotators and MMM during locomotion and across all behavioral classes. **d** Ethogram of grooming modes produced from MMM and individual annotators. **e** Quantification of the agreement overlap between individual annotators and MMM during face/paw grooming and across all grooming modes.

**Additional file 4: Fig. S4.** Behavioral summary of C57BL/6J male and female mice. **a** Behavioral usage for each of eight coarse categories plotted for C57BL/6J male(top) and female (bottom) mice for each of five observation days. All individuals are shown as points, colored traces correspond to the median fraction of time spent in the behavior for each day. **b** The mean usage of each coarse behavioral class during 20 min of observation for each of five days. Shaded regions represent 95% confidence interval.

**Additional file 5: Fig. S5.** Behavioral summary of Cntnap2 KO mice. **a** Behavioral usage for each of eight coarse categories plotted for Cntnap2 KO WT (top), heterozygote (middle) and homozygote (bottom) mice for each of four observation days. All individuals are shown as points, colored traces correspond to the median fraction of time spent in the behavior for each day. **b** The mean usage of each coarse behavioral class during 20 min of observation for each of four days. Shaded regions represent 95% confidence interval.

**Additional file 6: Fig. S6.** Behavioral summary of L7-Tsc1 mutant mice. **a** Behavioral usage for each of eight coarse categories plotted for L7-Tsc1 mutant WT (top), heterozygote (middle) and homozygote (bottom) mice for each of four observation days. All individuals are shown as points, colored traces correspond to the median fraction of time spent in the behavior for each day. **b** The mean usage of each coarse behavioral class during 20 min of observation for each of four days. Shaded regions represent 95% confidence interval.

**Additional file 7: Fig. S7.** Summary of behavioral phenotypes of experimental groups.

## Data Availability

All data required to reproduce results are available at Princeton Library Research Databases. All code for the mouse LEAP models and accompanying metrics are packed with skeleton and labeling data. Raw behavior movies are available upon request. Database with time series of predicted positions of virtual markers, training dataset and PCA components can be downloaded at digital DataSpace repository https://doi.org/10.34770/bzkz-j672. The Github repository https://github.com/PrincetonUniversity/MouseMotionMapper is set up to use the batch processing system Slurm and contains the code to reproduce the analysis pipeline. The repository contains code for reproducing all figures in the main text and supplement. Examples of preprocessing steps, unsupervised classification, reembedding, fingerprinting and all other utility functions used are also included.

## Abbreviations

ASD: Autism Spectrum Disorders
Cntnap2: Contactin-Associated Protein 2
KO: gene-knockout
L7-Tsc1: Purkinje cell-specific mouse model of tuberous sclerosis complex 1
LEAP: deep learning-based framework for estimating positions of animal body parts
tSNE: t-distributed stochastic neighbor embedding
