# Supplementary figures and images for: Deep phenotyping reveals movement phenotypes in mouse neurodevelopmental models
Source: Mol Autism. 2022 Mar 12;13:12. doi: 10.1186/s13229-022-00492-8 (PMC8917660; doi:10.1186/s13229-022-00492-8)

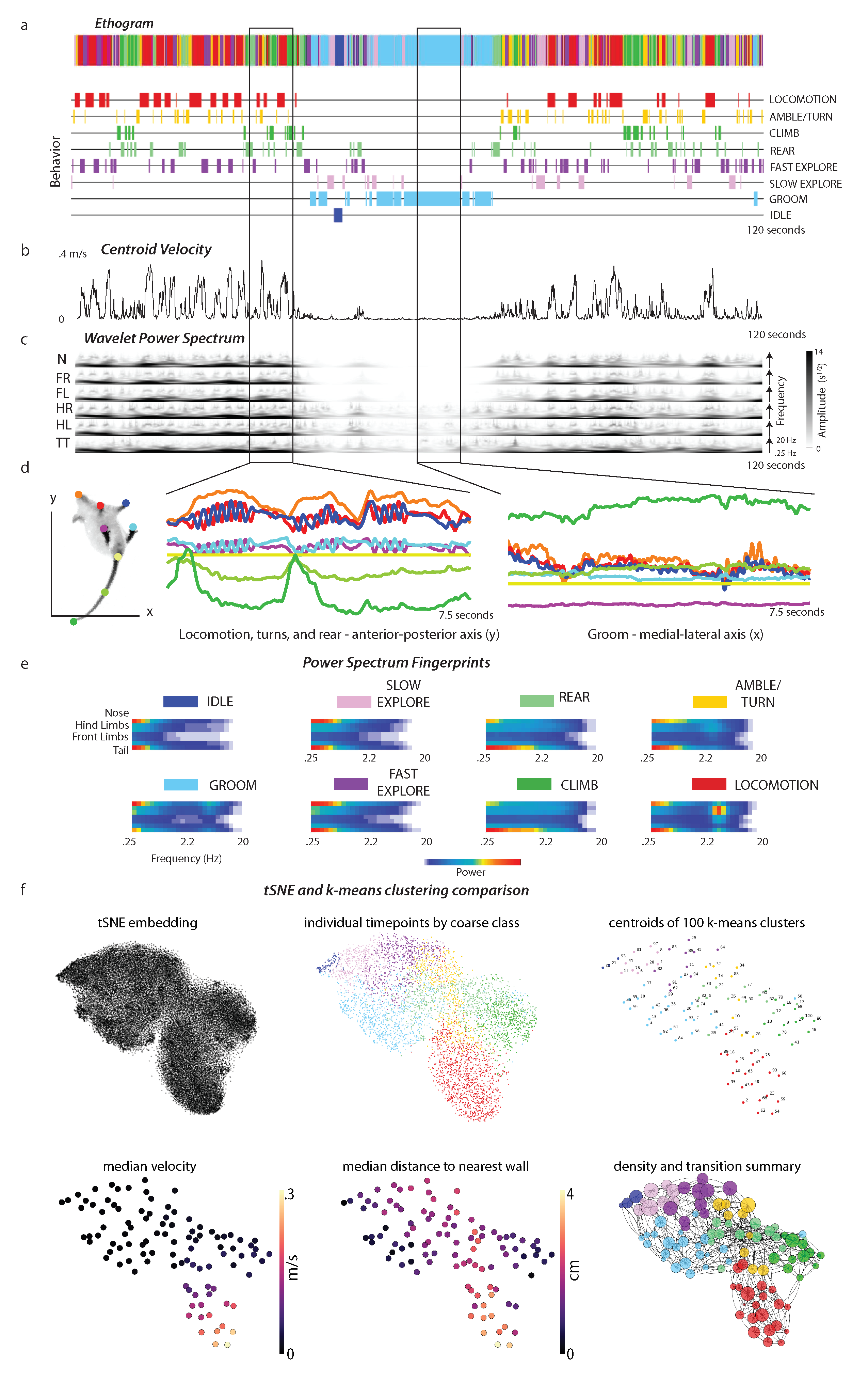

Supplement: Supplementary file 1 — Additional file 1: Fig. S1. a Ethogram of behavioral classes produced from our behavioral clustering and classification pipeline based on the posture and movement of animals over time. Below, the same time series is visualized as a raster to demonstrate behavioral usage during a 2-min period. b Mouse centroid speed over the same 2-min period shown in panel (a). c Raw power for select body parts. N: nose, FR: front right foot, FL: front left foot, HR: hind right foot, HL: hind left foot, TT: tail tip. d Position time series for the nose, front and hind feet, and tail tip (left) during two behaviors. The y position, the anterior–posterior axis, is shown for a locomotion bout (center). The x position, the medial–lateral axis, is shown for a grooming bout (right). We use the full 2D position of each body part in our analyses, but show only the dominant axis for these behaviors here for brevity. e Normalized power spectra for several tracked body parts for each of the eight behavior classes. f Visualizations of the tSNE embedding for a subselection of data show how the methods are related. [file 13229_2022_492_MOESM1_ESM.tif]

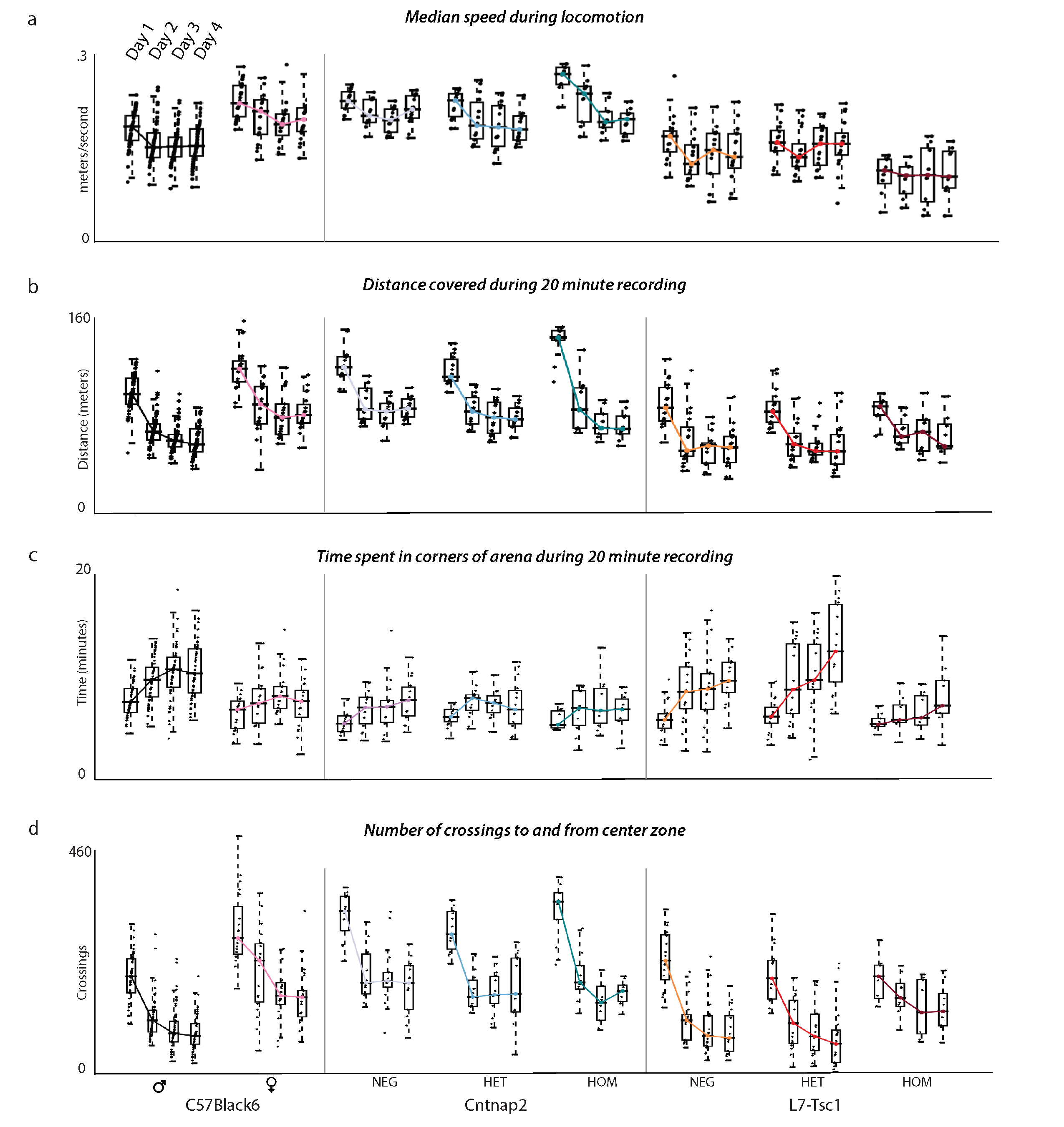

Supplement: Supplementary file 2 — Additional file 2: Fig. S2. Common metrics for open-field performance plotted for all conditions. a Median velocity during locomotion, b Median distance covered during 20 min in the open field. c The total time spent in the corner regions for each mouse on each day displayed as a box plot. The median value is shown as a solid line in the color corresponding to the given condition. d The number of center crossings for each mouse on each day displayed as a box plot. The median value is shown as a solid line in the color corresponding to the given condition. [file 13229_2022_492_MOESM2_ESM.tif]

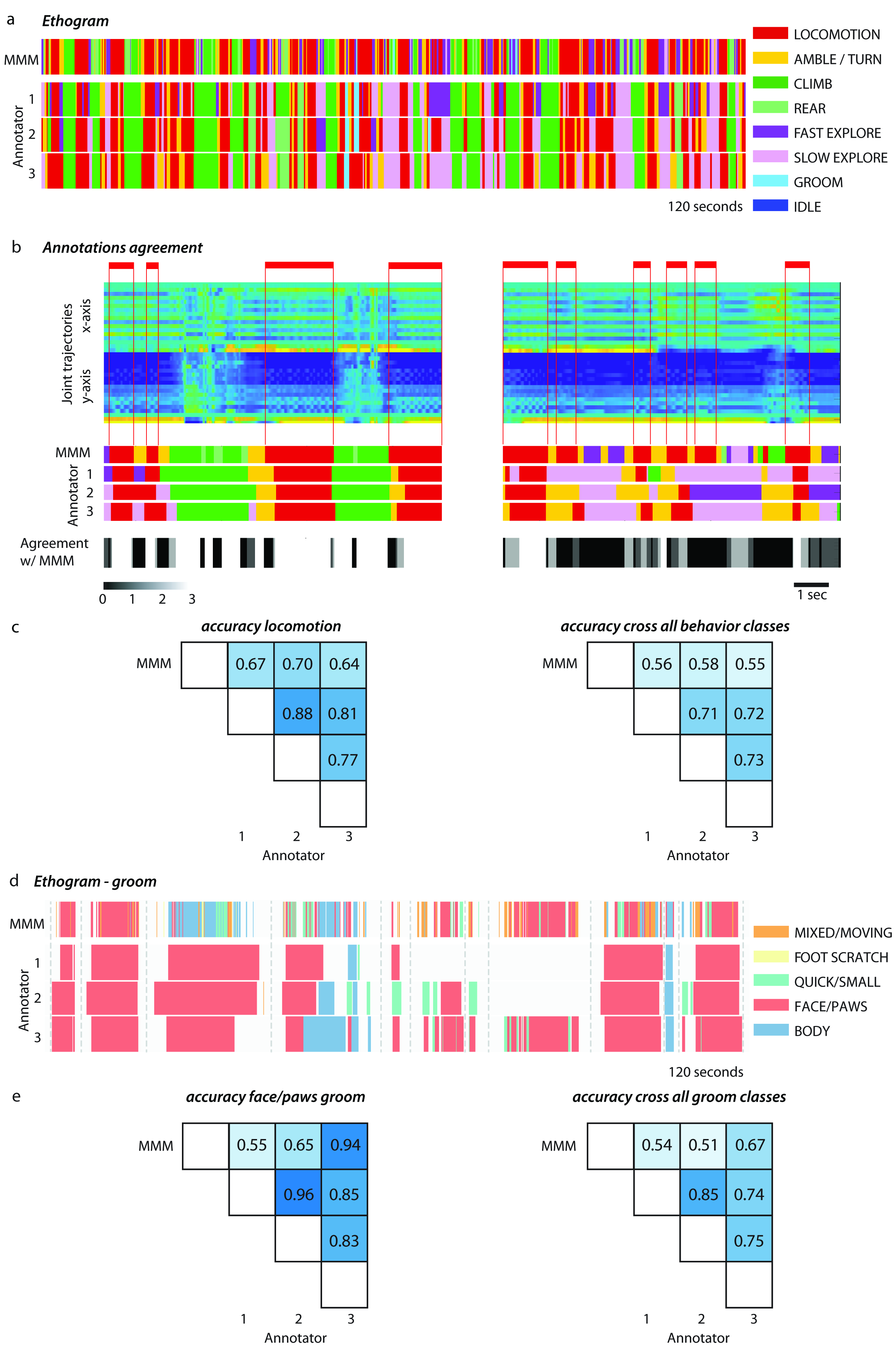

Supplement: Supplementary file 3 — Additional file 3: Fig. S3. Annotation of mouse behavioral classes. a Ethogram of behavioral classes produced from MouseMotionMapper (MMM) and individual annotators. b Typical agreements and discrepancies between MMM and human annotators synchronized with body parts trajectories. c Quantification of the agreement overlap between individual annotators and MMM during locomotion and across all behavioral classes. d Ethogram of grooming modes produced from MMM and individual annotators. e Quantification of the agreement overlap between individual annotators and MMM during face/paw grooming and across all grooming modes. [file 13229_2022_492_MOESM3_ESM.tif]

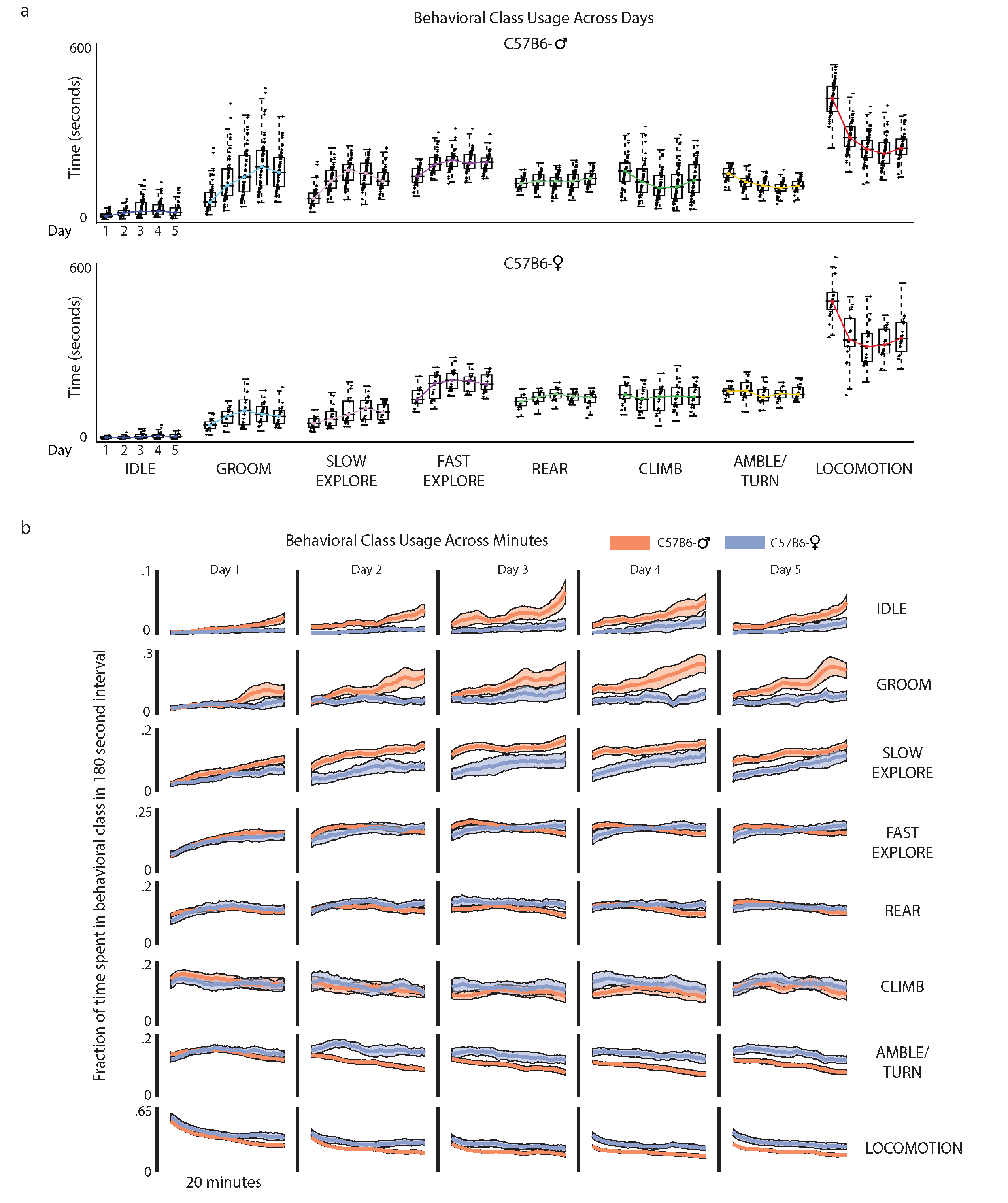

Supplement: Supplementary file 4 — Additional file 4: Fig. S4. Behavioral summary of C57BL/6J male and female mice. a Behavioral usage for each of eight coarse categories plotted for C57BL/6J male(top) and female (bottom) mice for each of five observation days. All individuals are shown as points, colored traces correspond to the median fraction of time spent in the behavior for each day. b The mean usage of each coarse behavioral class during 20 min of observation for each of five days. Shaded regions represent 95% confidence interval. [file 13229_2022_492_MOESM4_ESM.tif]

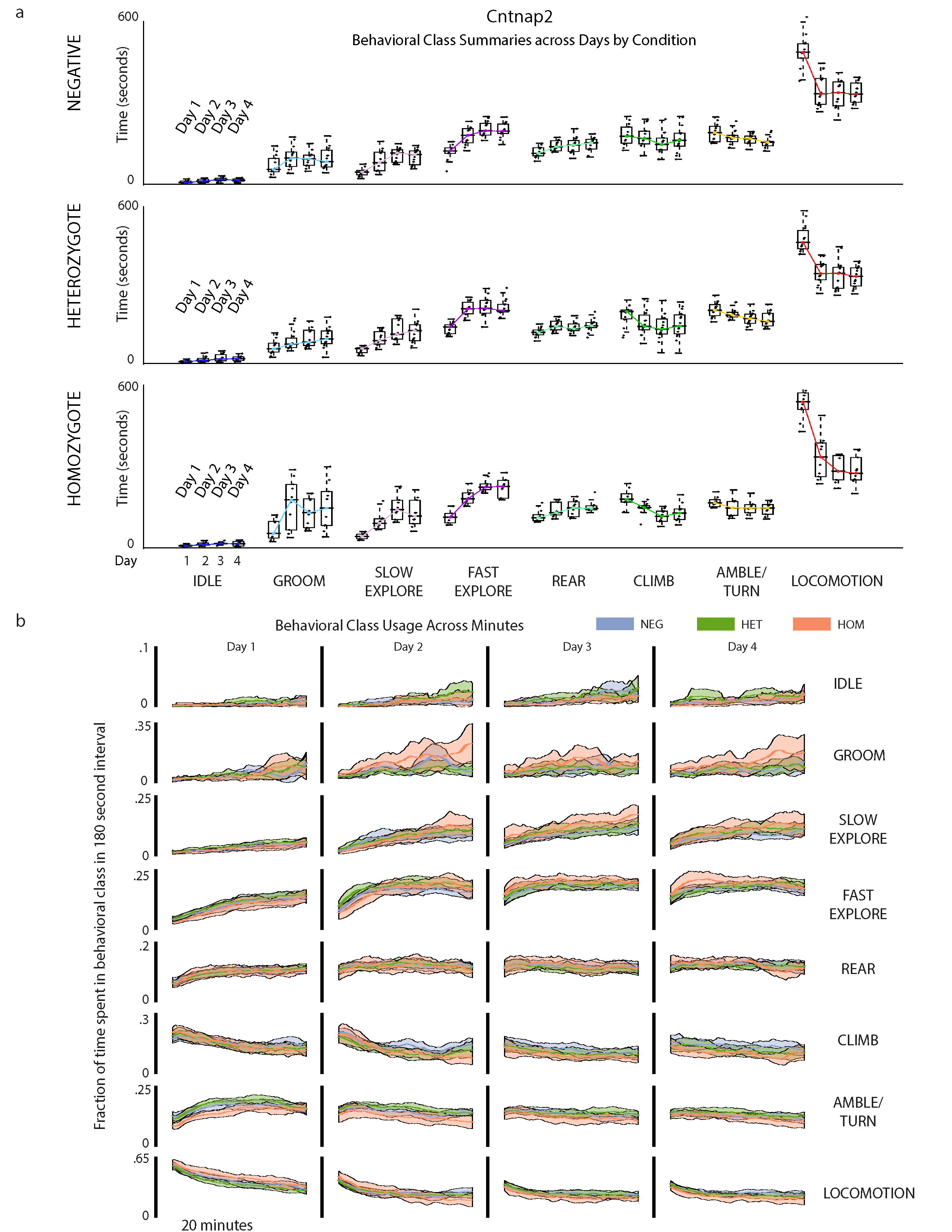

Supplement: Supplementary file 5 — Additional file 5: Fig. S5. Behavioral summary of Cntnap2 KO mice. a Behavioral usage for each of eight coarse categories plotted for Cntnap2 KO WT (top), heterozygote (middle) and homozygote (bottom) mice for each of four observation days. All individuals are shown as points, colored traces correspond to the median fraction of time spent in the behavior for each day. b The mean usage of each coarse behavioral class during 20 min of observation for each of four days. Shaded regions represent 95% confidence interval. [file 13229_2022_492_MOESM5_ESM.tif]

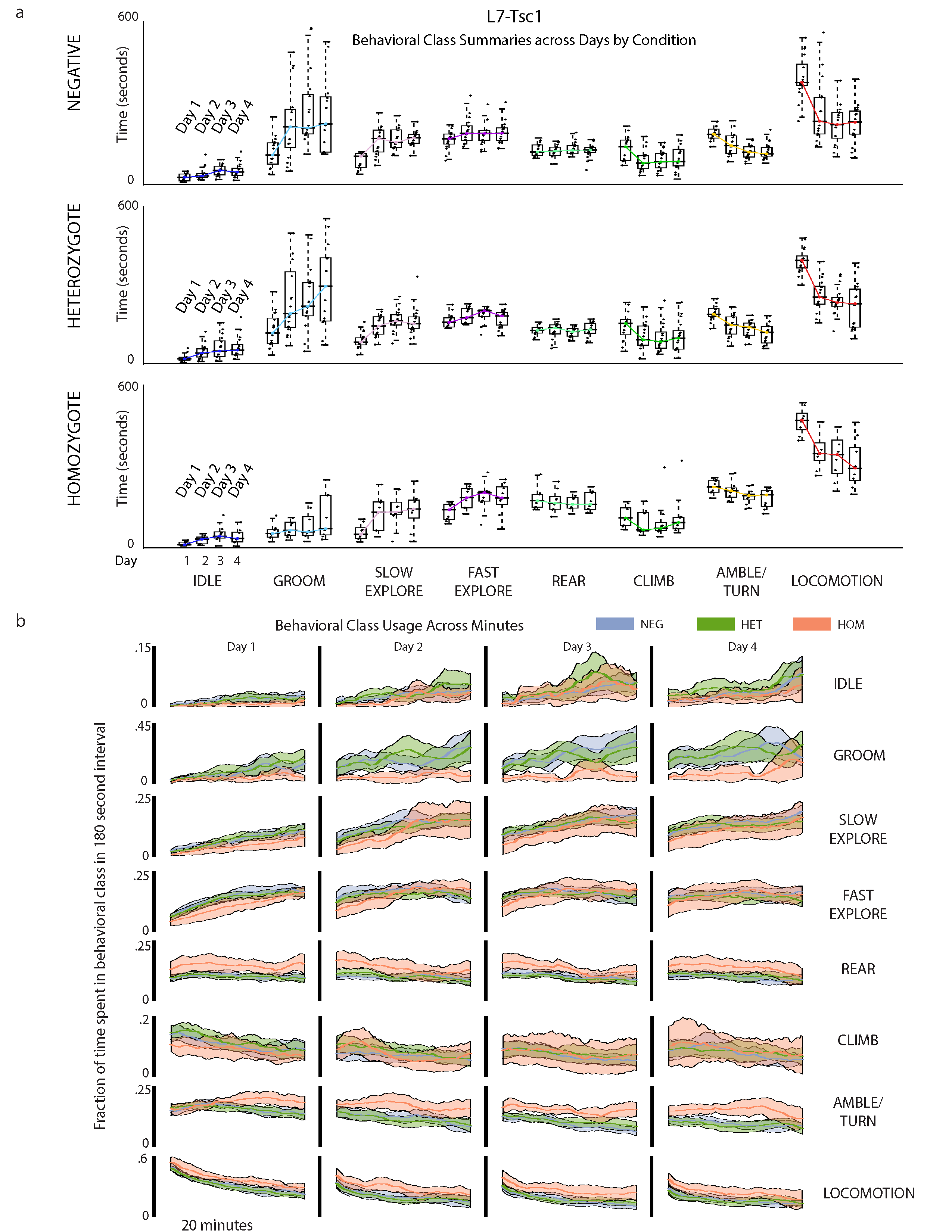

Supplement: Supplementary file 6 — Additional file 6: Fig. S6. Behavioral summary of L7-Tsc1 mutant mice. a Behavioral usage for each of eight coarse categories plotted for L7-Tsc1 mutant WT (top), heterozygote (middle) and homozygote (bottom) mice for each of four observation days. All individuals are shown as points, colored traces correspond to the median fraction of time spent in the behavior for each day. b The mean usage of each coarse behavioral class during 20 min of observation for each of four days. Shaded regions represent 95% confidence interval. [file 13229_2022_492_MOESM6_ESM.tif]

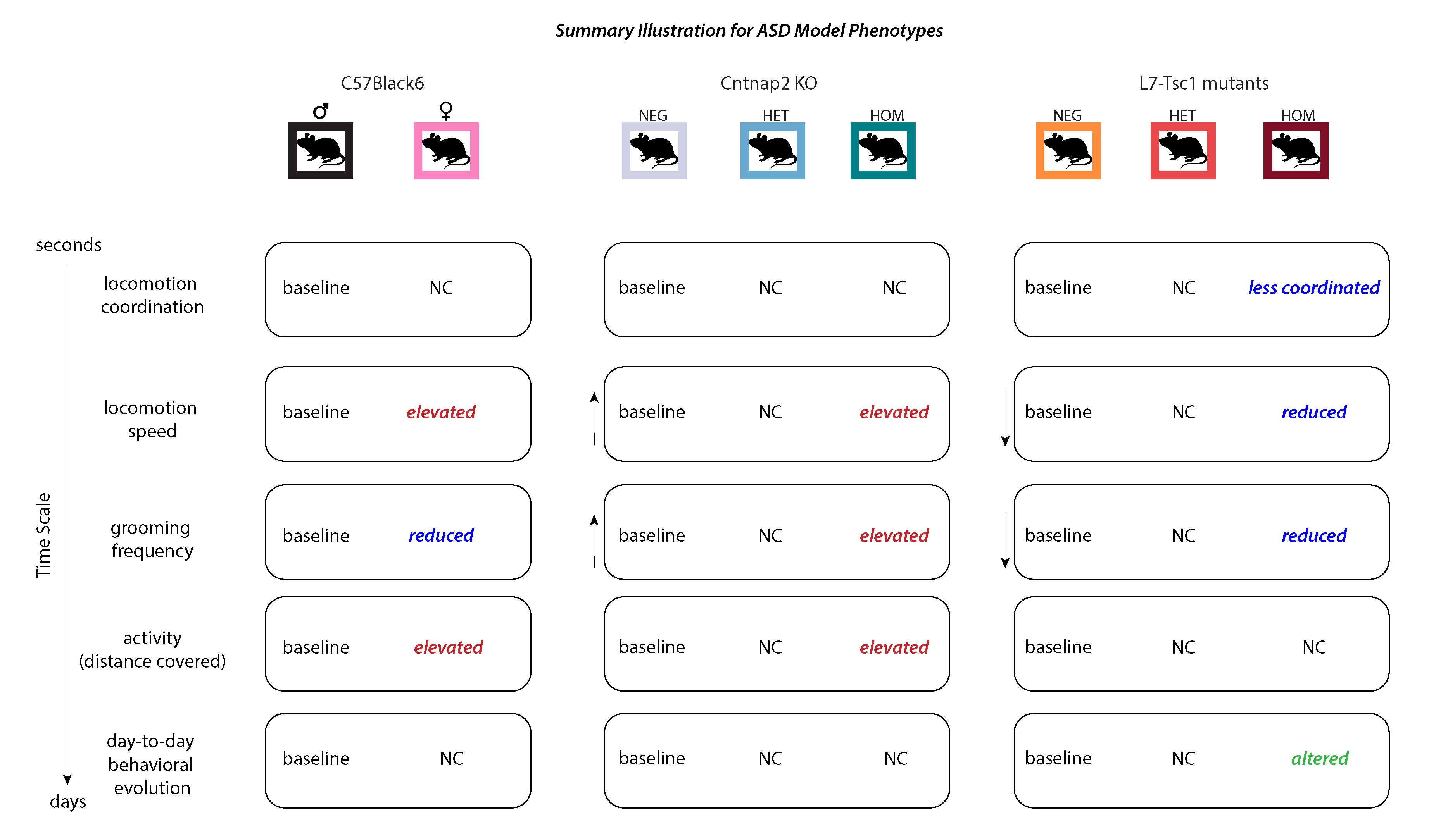

Supplement: Supplementary file 7 — Additional file 7: Fig. S7. Summary of behavioral phenotypes of experimental groups. [file 13229_2022_492_MOESM7_ESM.tif]
